# Prognostic Value of D-dimer in patients with acute coronary syndrome treated by percutaneous coronary intervention: a retrospective cohort study

**DOI:** 10.1186/s12959-021-00281-y

**Published:** 2021-05-07

**Authors:** Runzhen Chen, Chen Liu, Peng Zhou, Yu Tan, Zhaoxue Sheng, Jiannan Li, Jinying Zhou, Yi Chen, Li Song, Hanjun Zhao, Hongbing Yan

**Affiliations:** 1 Fuwai Hospital, Chinese Academy of Medical Sciences, Shenzhen, China; 2 Fuwai Hospital, National Center for Cardiovascular Diseases, Peking Union Medical College, Chinese Academy of Medical Sciences, 167 Beilishi Rd, Xicheng District, 100037 Beijing, China; 3 Xiamen Cardiovascular Hospital, Xiamen University, Xiamen, China; 4 China-Japan Friendship Hospital, Beijing, China

**Keywords:** D-dimer, Acute coronary syndromes, Percutaneous coronary intervention, Prognosis, Risk stratifications

## Abstract

**Background:**

Associations between D-dimer and outcomes of patients with acute coronary syndromes (ACS) remain controversial. This study aimed to investigate the prognostic value of D-dimer in ACS patients treated by percutaneous coronary intervention (PCI).

**Methods:**

In this observational study, 3972 consecutive patients with ACS treated by PCI were retrospectively recruited. The X-tile program was used to determine the optimal D-dimer thresholds for risk stratifications. Cox regression with multiple adjustments was used for outcome analysis. Restricted cubic spline (RCS) analysis was performed to assess the dose-response association between D-dimer and outcomes. The C-index was calculated to evaluate the additional prognostic value of D-dimer when added to clinical risk factors and commonly used clinical risk scores, with internal validations using bootstrapping methods. The primary outcome was all-cause death.

**Results:**

During a median follow-up of 720 days, 225 deaths occurred. Based on the thresholds generated by X-tile, ACS-PCI patients with median (420–1150 ng/mL, hazard ratio [HR]: 1.58, 95 % confidence interval [CI]: 1.14–2.20, *P* = 0.007) and high (≥ 1150 ng/mL, HR: 1.98, 95 % CI: 1.36–2.89, *P* < 0.001) levels of D-dimer showed substantially higher risk of death compared to those with low D-dimer (< 420 ng/mL). RCS analysis depicted a constant relation between D-dimer and various outcomes. The addition of D-dimer levels significantly improved risk predictions for all-cause death when combined with the fully adjusted models (C-index: 0.853 vs. 0.845, P _difference_ = 0.021), the GRACE score (C-index: 0.826 vs. 0.814, P _difference_ = 0.027), and the TIMI score (C-index: 0.804 vs. 0.776, P _difference_ < 0.001). The predicted mortality at the median follow-up (two years) was 1.7 %, 5.2 %, and 10.9 % for patients with low, median, and high D-dimer, respectively, which was well matched with the observed mortality (low D-dimer group: 1.2 %, median D-dimer group: 5.2 %, and high D-dimer group: 12.6 %).

**Conclusions:**

For ACS patients treated by PCI, D-dimer level was an independent predictor for adverse outcomes, and provided additional prognostic value when combined with clinical risk factors and risk scores. Risk stratifications based on D-dimer was plausible to differentiate ACS-PCI patients with higher risk of death.

**Supplementary Information:**

The online version contains supplementary material available at 10.1186/s12959-021-00281-y.

## Background

D-dimer, the direct product from degradation of cross-linked fibrin, was one of the most widely used biomarkers for diagnosis and outcomes predictions of thrombotic and vascular diseases [1–6]. Recent studies show that D-dimer improves the risk predictions among patients with stable coronary heart disease treated by percutaneous coronary intervention (PCI) [3, 4]. Basically, the elevation of D-dimer reflects the activation of coagulation and fibrinolysis, which indicates a systemic prothrombotic state [1, 7]. Evidences from intravascular imaging studies also suggest a positive linkage between D-dimer level and vulnerability of atherosclerotic plaques [8, 9]. Therefore, D-dimer could be a valuable biomarker for detecting hypercoagulation and unstable lesions, which are important pathogenesis for acute coronary events [10, 11], and might have the potential to assist risk stratifications of patients with coronary heart disease.

However, outcome predictions based on D-dimer have been seldom used in current clinical practice, as there are no clear definitions of high-risk populations. The cut-off thresholds for risk stratifications have not yet been developed or validated, as most previous studies generally divide patients according to selected percentiles [3, 4, 12, 13]. It is also not clear whether the risk of adverse outcomes grow constantly along with the increase of D-dimer, since there is no study analyzing the dose-response association between D-dimer and outcomes. Moreover, the prognostic value of D-dimer remains controversial in the context of acute coronary syndrome (ACS), as previous studies report huge variation regarding hazards associated with D-dimer elevation, and its value for improving risk predictions [12–18]. Therefore, this study aimed to investigate the followings: (1) the association between D-dimer and adverse outcomes, (2) the optimal D-dimer cut-off thresholds for risk stratifications, and (3) whether D-dimer could improve the risk predictions when added to common clinical risk factors and commonly used clinical risk scores, in order to offer evidence for clinical applications of D-dimer in ACS patients.

## Methods

### Study cohort

This retrospective study was based on a prospective cohort in a large-volume PCI center at a national tertiary care institute (Fuwai Hospital, Beijing) specializing in cardiovascular diseases, which has enrolled all patients undergoing coronary angiography and PCI procedures (emergent or selective) from January 2010 to June 2017. ACS consisted of ST-segment elevation myocardial infarction (STEMI) and non-ST-elevation ACS (NSTE-ACS). Diagnoses and classifications of ACS were made according to guidelines and universal definitions up to date, including criteria of clinical presentations, typical characteristics on electrocardiography, dynamical changes of cardiac enzymes, and imaging evidence [19–21]. All patients diagnosed with ACS subsequently undergoing emergent coronary angiography and PCI were included in this study. Patients were excluded if they had thrombotic diseases diagnosed during the index hospitalizations (i.e., pulmonary embolism, deep vein thrombosis, arterial thrombosis, mural thrombus, and thrombophlebitis), or no follow-up records. The study was performed in accordance with principles set forth in the Declaration of Helsinki, and was approved by the ethics committee of the institute. All patients had signed the written informed consents during hospitalization regarding the use of clinical data for the purpose of scientific research by the institute.

### Coronary angiography and PCI procedure

Emergent coronary angiography and PCI procedures were routinely performed through radial access. Heparin (100 IU/kg) was routinely administered during the procedures. Blood flow of the infarct-related artery was assessed according to Thrombolysis in Myocardial Infarction (TIMI) grading system. The use of certain PCI techniques and relevant devices (e.g., stenting, balloon dilation, thrombus aspiration, intra-aortic balloon pump) were at the discretion by the operators. All patients undergoing emergent PCI were subsequently monitored in the coronary care unit.

### The clinical risk scores

The calculation for the Global Registry of Acute Coronary Events (GRACE) score and the TIMI score to predict mortality has been described in details by earlier researches [22–24]. Clinical data required for calculating the risk scores were retrieved from patient medical records, including age, weight, established clinical risk factors (diabetes, hypertension, and history of angina), systolic blood pressure, heart rate, creatinine level, congestive heart failure, presence of cardiac arrest, elevation of cardiac enzymes, ST-segment deviation on the electrocardiogram, anterior ST elevation or left bundle branch block, and time to treatment.

### Laboratory measurements

After the PCI procedure, blood samples for D-dimer tests were routinely collected via the cubital vein as soon as patients were admitted into the coronary care unit. Plasma D-dimer level was obtained using immunoassay turbidimetry (STA Compact Assay Instrument, Diagnostica Stago Inc, France). Blood samples for other biometrics tests were collected at 6 a.m. on the next morning, while cardiac enzymes were tested on a daily basis or as required by physicians in charge.

### Outcomes and follow‐up

The primary outcome for the current analysis was all-cause death. The secondary outcomes included the followings: (1) cardiac death; (2) major adverse cardiovascular event (MACE), which was a composite of all-cause death, recurrent myocardial infarction (MI), and ischemic stroke. Patients would be routinely followed up at 1, 6, and 12 months after discharge. The follow-up was completed independently by staffs from the information center of the institute, using standardized questionnaires through phone-call interview, and the outcome data was then transferred to the research group on a monthly basis. Follow-up was also performed during rehospitalizations and outpatient visits at the institute because of adverse events or re-examinations. For those who survived more than a year, the subsequent follow-up would be made annually. A group of physicians (R. Chen, C. Liu, and J. Zhou) routinely assessed the reported adverse events. In case of a dispute, a consensus was reached by discussions.

### Statistical analysis

All the statistical analysis was performed using Stata 15.0 (Stata Corp, College Station, TX, USA) and R 3.6.0 (R Core Team, Vienna, Austria). Categorial variables are presented as numbers (%). Continuous variables are presented using mean ± SD if they follow the normal distribution. Otherwise, they are presented as medians with the 25th and 75th percentiles. Multiple imputations were performed for missing values of lab test results using the *mi* command. The X-tile program (Rimm Lab, Yale School of Medicine) was used to find the optimal cut-off thresholds of D-dimer to differentiate patients into high, median, and low risk groups. Univariable Cox regression model was used to assess the association between D-dimer and clinical outcomes, followed by multivariable adjustments. The models were adjusted for established risk factors of ACS patients [19, 20], including age, sex, diabetes mellitus, hypertension, peripheral artery disease, history of coronary artery bypass graft or PCI, diagnosis of STEMI, incidence of cardiac arrest, ejection fraction, low-density lipoprotein cholesterol, high-sensitivity C-reactive protein, creatinine, peak level of cardiac troponin I, culprit lesion, multivessel disease, pre- and post-procedure TIMI grade flow, door-to-balloon time, stent placements, complete revascularization before discharge, and the use of aspirin, P2Y12 inhibitors and statins. Models fully adjusted for all collected baseline variables, the GRACE score, and the TIMI score were also performed. To assess the dose-response association between D-dimer and outcomes, logarithmic D-dimer was entered into the model adjusted for all baseline variables using restrictive cubic spline (RCS), in order to avoid nonlinearity. To evaluate the additional prognostic value of D-dimer when combined with clinical risk factors and risk scores, C-index was calculated and compared as described previously [25], using bootstrapping of 1000 replications for internal validation. The calibration curve was used to analyze the agreement between model predictions and actual observations. A *P*-value < 0.05 was considered statistically significant.

## Results

### Patient cohort and baseline characteristics

From January 2010 to June 2017, the institute had admitted 4151 patients who underwent emergent coronary angiography and PCI due to ACS. Among these patients, 86 (2.1 %) patients were diagnosed with thrombotic diseases during the index hospitalization, and 93 (2.2 %) patients did not have follow-up records of phone-call interview, outpatient visits or re-hospitalization at the institute. Finally, a total of 3972 (95.7 %) patients were included in the current analysis. The mean age of included patients was 59.0 ± 11.9 years, and 78.8 % of the patients were male (Table 1). Overall, the level of post-procedural D-dimer was 330 (220–590) ng/mL (fibrinogen-equivalent units). Patients who died during the follow-up tended to be older, female, and complicated with more comorbidities and worse cardiac function. Their D-dimer level was significantly higher than those who survived, along with substantially higher level of high-sensitivity C-reactive protein, creatinine and cardiac troponin I. Culprit lesions of left main artery, three-vessel disease and worse anterograde blood flow were more commonly seen among patients failing to survive, and they tended to receive fewer coronary stentings or oral medications, but more placements of intra-aortic balloon pump. During a median follow-up of 720 (428–2054) days, there were 225 deaths (5.7 %, incidence rate [IR]: 17.6/1000-person-years [PY]), 151 cardiac deaths (3.8 %, IR: 11.8/1000 PY), and 408 MACEs (10.3 %, IR: 31.9/1000 PY) in total.

**Table 1:** Baseline characteristics of study patients stratified by the primary outcome

	Overall (*N* = 3972)	Survivors (*N* = 3747)	Non-survivors (*N* = 225)	*P*-value
Age, years	59.0 ± 11.9	58.4 ± 11.8	68.4 ± 10.6	< 0.001
Male sex, n (%)	3132 (78.8)	2993 (79.9)	139 (61.8)	< 0.001
Diabetes mellitus, n (%)	1299 (32.7)	1207 (32.2)	92 (40.9)	0.007
Hypertension, n (%)	2429 (61.2)	2262 (60.4)	167 (74.2)	< 0.001
Peripheral artery diseases, n (%)	159 (4.0)	141 (3.8)	18 (8.0)	0.002
Previous CABG or PCI, n (%)	583 (14.7)	548 (14.6)	35 (15.6)	0.702
STEMI, n (%)	3467 (87.3)	3269 (87.2)	198 (88.0)	0.741
Tumor diseases, n (%)	81 (2.0)	71 (1.9)	10 (4.4)	0.009
Liver diseases, n (%)	570 (14.8)	555 (14.8)	15 (6.7)	0.001
COPD, n (%)	32 (0.8)	27 (0.7)	5 (2.2)	0.014
Hemodynamics				
Heart rate, bpm	77.4 ± 15.2	77.0 ± 14.9	83.4 ± 19.7	< 0.001
Systolic blood pressure, mmHg	124.7 ± 18.3	124.8 ± 18.1	123.0 ± 21.9	0.157
EF, %	53.8 ± 7.6	54.1 ± 7.4	48.6 ± 9.2	< 0.001
Cardiac arrest, n (%)	144 (3.6)	125 (3.3)	19 (8.4)	< 0.001
Laboratory tests				
D-dimer, ng/mL	330 (220–590)	330 (220–550)	600 (320–1240)	< 0.001
LDL-C, mmol/L	2.7 ± 0.9	2.7 ± 0.9	2.7 ± 0.9	0.331
hsCRP, mg/L	7.16 (2.75–12.03)	7.00 (2.71–11.94)	11.17 (3.88–13.13)	< 0.001
Creatinine, µmoI/L	82.0 ± 25.1	81.0 ± 22.8	98.1 ± 47.1	< 0.001
Peak cTnI, ng/mL	2.50 (0.44–10.80)	2.48 (0.44–10.62)	2.97 (0.62–14.01)	0.093
GRACE score	109.7 ± 27.7	107.7 ± 25.9	142.0 ± 35.0	< 0.001
TIMI score	6.3 ± 1.8	6.2 ± 1.8	8.0 ± 2.0	< 0.001
Findings and details of PCI procedures				
Culprit lesion, n (%)				
Left main artery	95 (2.4)	80 (2.1)	15 (6.7)	< 0.001
Left anterior descending artery	1734 (43.7)	1632 (43.6)	102 (45.3)	
Left circumflex	610 (15.4)	592 (15.8)	18 (8.0)	
Right coronary artery	1515 (38.1)	1429 (38.1)	86 (38.2)	
Bypass graft	18 (0.5)	14 (0.4)	4 (1.8)	
Multi-vessel disease, n (%)				
1-vessel disease	1002 (25.2)	966 (25.8)	36 (16.0)	< 0.001
2-vessel disease	1253 (31.6)	1201 (32.1)	52 (23.1)	
3-vessel disease	1717 (43.2)	1580 (42.2)	137 (60.9)	
Pre-PCI TIMI 0 flow, n (%)	2607 (65.6)	2441 (65.2)	166 (73.8)	0.008
Post-PCI TIMI 3 flow, n (%)	3822 (96.2)	3617 (96.5)	205 (91.1)	< 0.001
D2B time, mins	128 (95–202)	128 (95–201)	125 (90–219)	0.342
Stent placements, n (%)	3497 (88.0)	3321 (88.6)	176 (78.2)	< 0.001
Thrombus aspiration, n (%)	1649 (41.5)	1561 (41.7)	88 (39.1)	0.451
IABP, n (%)	381 (9.6)	323 (8.6)	58 (25.8)	< 0.001
Glycoprotein IIb/IIIa inhibitor, n (%)	534 (13.4)	507 (13.5)	27 (12.0)	0.513
Complete revascularization before discharge, n (%)	1731 (43.6)	1669 (44.5)	62 (27.6)	< 0.001
Medications ^a^				
Aspirin, n (%)	3930 (98.9)	3718 (99.2)	212 (94.2)	< 0.001
P2Y12 inhibitors, n (%)	3936 (99.1)	3719 (99.3)	217 (96.4)	< 0.001
Statins, n (%)	3708 (93.4)	3507 (93.6)	201 (89.3)	0.013

^a^ Medication typically referred to drugs prescribed at discharge, or otherwise, drugs being used during hospitalization if patients failed to survive the hospitalization. bpm = beats per minutes, CABG = Coronary artery bypass grafting, COPD = chronic obstructive pulmonary diseases, cTnI = cardiac troponin I, D2B time = door-to-balloon time, EF = ejection fraction, GRACE score = the Global Registry of Acute Coronary Events risk score, hsCRP = high sensitivity C-reactive protein, IABP = intra-aortic balloon pump, LDL-C = low-density lipoprotein cholesterol, PCI = percutaneous coronary intervention, STEMI = ST-segment elevation myocardial infarction, TIMI flow = the Thrombolysis In Myocardial Infarction grade flow, TIMI score = the Thrombolysis In Myocardial Infarction risk score

### Associations between D-dimer and outcomes

**Fig. 1:**
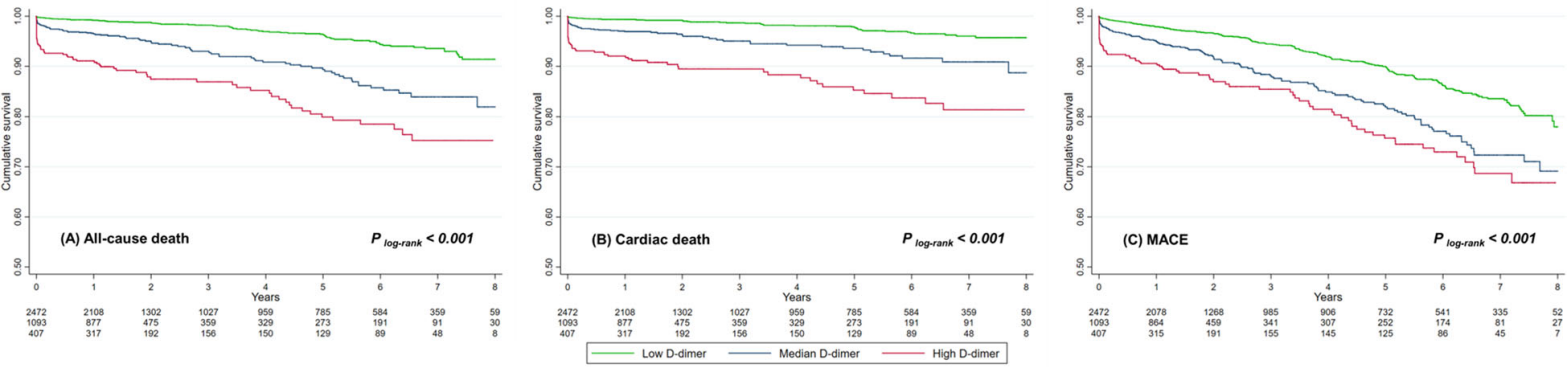
Kaplan-Meier survival curve analysis for all-cause death (**a**), cardiac death (**b**), and major adverse cardiovascular events (MACE) according to low (< 420 ng/mL), median (420–1150 ng/mL), and high (≥ 1150 ng/mL) levels of D-dimer

**Fig. 2:**
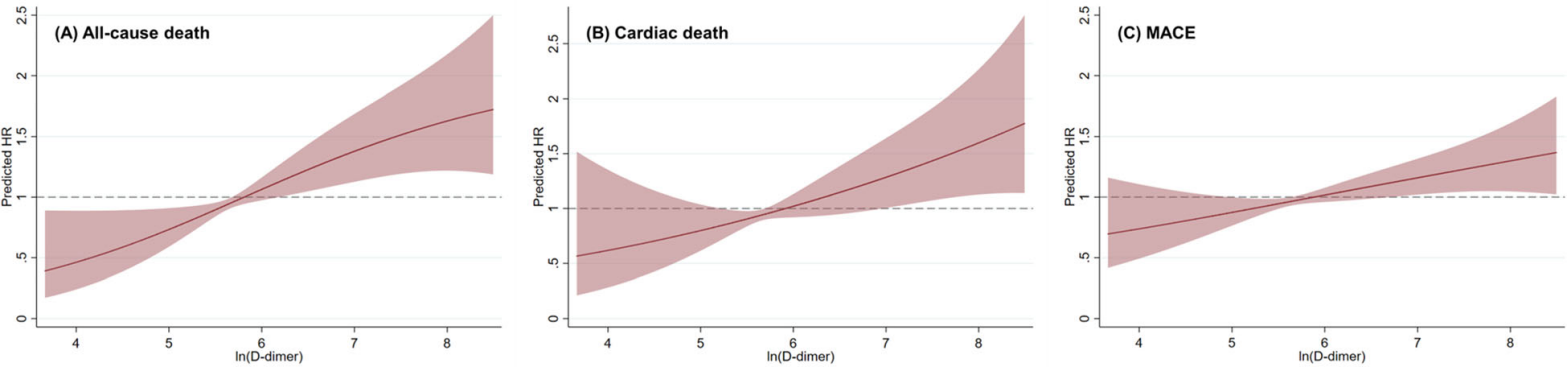
Continuous hazard ratio across logarithmic D-dimer for all-cause death (**a**), cardiac death (**b**), and major adverse cardiovascular events (**c**) according to restricted cubic spline analysis. HR = hazard ratio, line = predicted HR, dashed area = 95 % confidence interval

According to the cut-off thresholds derived from the X-tile program, 2472 (62.2 %) patients were classified into low D-dimer group (< 420 ng/mL), 1093 (27.5 %) patients were classified into median D-dimer group (420–1150 ng/mL), and 407 (10.3 %) patients were classified into high D-dimer group (≥ 1150 ng/mL), respectively. The Kaplan-Meier analysis (Fig. 1) showed significant differences of survival among three groups of patients for both primary and secondary outcomes (all P _log−rank_ < 0.001). Associations between D-dimer levels and various outcomes are shown in Table 2. Univariable analysis showed that higher D-dimer levels were associated with increased mortality and incidence of MACE (Table 2). D-dimer levels remained as independent predictors of adverse events after adjustments for established risk factors, all baseline variables, the GRACE score and the TIMI score. In the fully-adjusted analysis (model 2), patients with median (420–1150 ng/mL, hazard ratio [HR]: 1.58, 95 % confidence interval [CI]: 1.14–2.20, *P* = 0.007) and high (≥ 1150 ng/mL, HR: 1.98, 95 % CI: 1.36–2.89, *P* < 0.001) levels of D-dimer showed substantially higher risk of all-cause death, as compared to those with low level of D-dimer (< 420 ng/mL). Significantly increased risk of cardiac death was also observed for patients with median (HR: 1.67, 95 % CI: 1.10–2.54, *P* = 0.016) and high (HR: 2.15, 95 % CI: 1.35–3.42, *P* = 0.001) levels of D-dimer. Similar increases of risk were also seen for the endpoint of MACE, where the relative risk increased for 26 % (*P* = 0.052) and 37 % (*P* = 0.038) for patients with median and high levels of D-dimer, respectively. For per unit increase of logarithmic D-dimer, the HRs were 1.26 (95 % CI: 1.10–1.44, *P* < 0.001) for all-cause death, 1.25 (95 % CI: 1.06–1.48, *P* = 0.008) for cardiac death, and 1.14 (95 % CI: 1.03–1.26, *P* = 0.013) for MACE, respectively. The RCS analysis showed that the increase of D-dimer was constantly associated with higher risk of both the primary and secondary endpoint events (Fig. 2).>

**Table 2:** Hazard ratio by D-dimer level for all-cause death, cardiac death, and major adverse cardiovascular events (MACE)

	Unadjusted HR (95 % CI)	*P*-value	Model 1 HR (95 % CI)	*P*-value	Model 2 HR (95 % CI)	*P*-value	Model 3 HR (95 % CI)	*P*-value	Model 4 HR (95 % CI)	*P*-value
All-cause death										
< 420 ng/mL	1 (reference)	-	1 (reference)	-	1 (reference)	-	1 (reference)	-	1 (reference)	-
420–1150 ng/mL	2.79 (2.04–3.80)	< 0.001	1.61 (1.16–2.23)	0.005	1.58 (1.14–2.20)	0.007	1.70 (1.24–2.34)	0.001	1.81 (1.32–2.49)	< 0.001
≥ 1150 ng/mL	5.33 (3.83–7.42)	< 0.001	2.09 (1.45–3.02)	< 0.001	1.98 (1.36–2.89)	< 0.001	2.46 (1.73–3.50)	< 0.001	3.33 (2.37–4.68)	< 0.001
Log (D-dimer)	1.68 (1.52–1.86)	< 0.001	1.28 (1.13–1.46)	< 0.001	1.26 (1.10–1.44)	0.001	1.36 (1.21–1.54)	< 0.001	1.51 (1.46–1.67)	< 0.001
Cardiac death										
< 420 ng/mL	1 (reference)	-	1 (reference)	-	1 (reference)	-	1 (reference)	-	1 (reference)	-
420–1150 ng/mL	2.91 (1.97–4.31)	< 0.001	1.17 (1.10–2.52)	0.016	1.67 (1.10–2.54)	0.016	1.67 (1.12–2.50)	0.012	1.82 (1.22–2.72)	0.004
≥ 1150 ng/mL	6.87 (4.61–10.23)	< 0.001	2.29 (1.46–3.60)	< 0.001	2.15 (1.35–3.42)	0.001	2.76 (1.80–4.24)	< 0.001	4.06 (2.69–6.12)	< 0.001
Log (D-dimer)	1.77 (1.57-2.00)	< 0.001	1.28 (1.09–1.50)	0.003	1.25 (1.06–1.48)	0.008	1.38 (1.19–1.60)	< 0.001	1.58 (1.37–1.82)	< 0.001
MACE										
< 420 ng/mL	1 (reference)	-	1 (reference)	-	1 (reference)	-	1 (reference)	-	1 (reference)	-
420–1150 ng/mL	1.86 (1.49–2.32)	< 0.001	1.29 (1.02–1.63)	0.031	1.26 (1.00-1.59)	0.052	1.38 (1.10–1.73)	0.005	1.44 (1.15–1.80)	0.002
≥ 1150 ng/mL	2.53 (1.94–3.29)	< 0.001	1.44 (1.08–1.92)	0.013	1.37 (1.02–1.83)	0.038	1.60 (1.23–2.11)	0.001	1.89 (1.44–2.47)	< 0.001
Log (D-dimer)	1.40 (1.29–1.52)	< 0.001	1.15 (1.05–1.27)	0.004	1.14 (1.03–1.26)	0.013	1.21 (1.10–1.33)	< 0.001	1.27 (1.16–1.40)	< 0.001

Model 1, adjusted for established risk factors (i.e. age, sex, hypertension, diabetes mellitus, peripheral artery diseases, history of CABG or PCI, diagnosis of STEMI, incidence of cardiac arrest, ejection fraction, LDL-C, hsCRP, creatinine, cardiac troponin I, culprit lesion, multivessel disease, pre- and post-procedure TIMI grade flow, door-to-balloon time, stent placements, complete revascularization before discharge, and the use of aspirin, P2Y_12_ inhibitors and statin); Model 2, adjusted for all collected baseline variables; Model 3, adjusted for the GRACE score; Model 4, adjusted for the TIMI score. CABG = coronary artery bypass grafting, CI = confidence interval, HR = hazard ratio, hsCRP = high-sensitivity C-reactive protein, LDL-C = low-density lipoprotein cholesterol, MACE = major adverse cardiovascular events, PCI = percutaneous coronary intervention, STEMI = ST-segment elevation myocardial infarction, TIMI flow = the Thrombolysis In Myocardial Infarction grade flow, TIMI score = the Thrombolysis In Myocardial Infarction risk score

### Improvement for risk predictions by D-dimer

According to C-index analyses with internal validation using bootstrapping methods, risk models established with clinical risk factors (C-index: 0.842, 95 % CI: 0.813–0.871, *P* < 0.001), all baseline variables (C-index: 0.845, 95 % CI: 0.818–0.872, *P* < 0.001), the GRACE score (C-index: 0.814, 95 % CI: 0.780–0.848, *P* < 0.001), and the TIMI score (C-index: 0.776, 95 % CI: 0.743–0.809, *P* < 0.001) all acquired favorable performance for predictions of all-cause death. However, the addition of D-dimer still brought significant increases in C-index to the four models above (Table 3). The C-index increased by 0.009 (95 % CI: 0.001–0.016, *P* = 0.019) for the model of established risk factors, 0.008 (95 % CI: 0.001–0.014, *P* = 0.021) for the model of all baseline variables, 0.012 (95 % CI: 0.001–0.024, *P* = 0.027) for the GRACE score, and 0.028 (95 % CI: 0.013–0.043) for the TIMI score. Similar improvement was observed when logarithmic D-dimer was added to existing models as continuous variables, but the C-index was slightly lower than that of corresponding models using stratified D-dimer levels. Calibration curve analyses were performed for 180-day, one-year, two-year, and five-year mortality, which showed good agreements of predictions and observations for all the above models including D-dimer (**Supplementary Figs.**1-8).

**Table 3:** Additional prognostic value of D-dimer for the primary outcome of all-cause death

	Risk factors ^a^	All baseline variables	GRACE score	TIMI score
**Original models**				
C-index	0.842 (0.813–0.871)	0.845 (0.818–0.872)	0.814 (0.780–0.848)	0.776 (0.743–0.809)
**Original models + D-dimer (high, median, low)**				
C-index	0.851 (0.823–0.879)	0.853 (0.826–0.879)	0.826 (0.794–0.859)	0.804 (0.773–0.835)
ΔC-index	0.009 (0.001–0.016)	0.008 (0.001–0.014)	0.012 (0.001–0.024)	0.028 (0.013–0.043)
P _difference_	0.019	0.021	0.027	< 0.001
**Original models + logarithmic D-dimer**				
C-index	0.849 (0.821–0.877)	0.851 (0.824–0.878)	0.825 (0.793–0.857)	0.800 (0.769–0.831)
ΔC-index	0.006 (0.001–0.012)	0.006 (0.001–0.011)	0.011 (0.002–0.020)	0.024 (0.011–0.037)
P _difference_	0.020	0.021	0.015	< 0.001

^a^ Risk factors model included age, sex, hypertension, diabetes mellitus, peripheral artery diseases, history of CABG or PCI, diagnosis of STEMI, incidence of cardiac arrest, ejection fraction, LDL-C, hsCRP, creatinine, cardiac troponin I, culprit lesion, multivessel disease, pre- and post-procedure TIMI grade flow, door-to-balloon time, stent placements, complete revascularization before discharge, and the use of aspirin, P2Y12 inhibitors and statin. CABG = coronary artery bypass graft, GRACE score = the Global Registry of Acute Coronary Events risk score, HR = hazard ratio, hsCRP = high-sensitivity C-reactive protein, LDL-C = low-density lipoprotein cholesterol, PCI = percutaneous coronary intervention, STEMI = ST-segment elevation myocardial infarction, TIMI grade flow = the Thrombolysis In Myocardial Infarction grade flow, TIMI score = the Thrombolysis In Myocardial Infarction risk score, ΔC-index = difference of C-index

### Risk stratifications by D-dimer

**Fig. 3:**
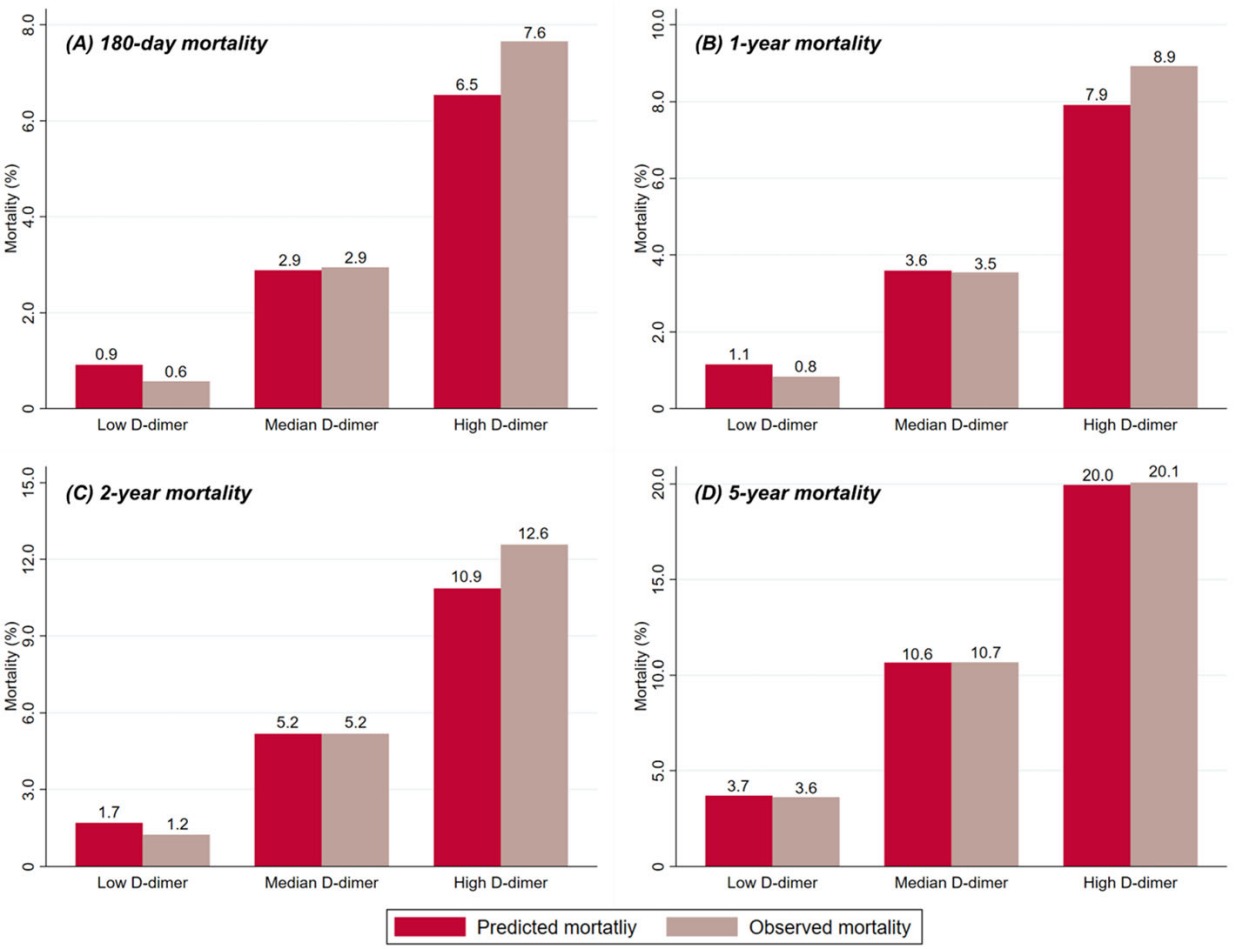
Predicted and observed mortality according to low (< 420 ng/mL), median (420–1150 ng/mL), and high (≥ 1150 ng/mL) levels of D-dimer at 180 days (**a**), one year (**b**), two years (**c**), and five years (**d**)

As risk models combining stratified D-dimer levels and all baseline variables showed the best performance, we calculated the predicted mortality at 180 days, one year, two year, and five years using this model, for which the results were compared with the actual observations (Fig. 3). The average predicted mortality at median follow-up (two years) was 1.7 %, 5.2 % and 10.9 % for patients with low, median, and high levels of D-dimer, respectively, which was well matched with the observed mortality (low D-dimer group: 1.2 %, median D-dime group: 5.2 %, and high D-dimer group: 12.6 %). The good correlation between predicted and observed mortality was also seen for shorter (180-day and one-year) and longer (five-year) follow-up.

## Discussions

The major findings of this study are as follow. For ACS patients treated by PCI: (1) D-dimer was an independent predictor for adverse outcomes, with the optimal cut-off thresholds at 420 ng/mL and 1150 ng/mL; (2) D-dimer level provided additional prognostic value when combined with established risk factors and clinical risk scores; (3) risk stratifications based on D-dimer could effectively differentiate patients with low, median, and high risk of all-cause death in both short and long term.

### D-dimer, mortality, and incidence of cardiovascular events

Previous research with small sample size reported conflicting results regarding the hazard of adverse events due to elevation of D-dimer. Akgul et al. report a 10.1-fold increase of 6-month mortality for STEMI patients undergoing primary PCI if D-dimer > 720 ng/mL [12]. Kikkert et al. report a 2.58-fold increase for risk of MACE if D-dimer ≥ 710 ng/mL in another STEMI cohort triaged to receive PCI, coronary artery bypass graft or medical therapy [15]. Erkol et al. suggest D-dimer loses its significance when adjusted for no-reflow phenomenon, but over 80 % of the patients were excluded in the final analysis due to unavailable D-dimer measurements [14]. Therefore, prognostic value of D-dimer remained controversial, as variation in target population, treating strategies and possible selection bias do not allow a consensus. Considering that PCI and invasive strategies are widely used for the whole spectrum of ACS, the current study focused on ACS patients treated by emergent PCI. Our results suggested a stable and constant dose-response association between D-dimer and the risk for various endpoint events according to continuous variable and RCS analysis. The median D-dimer level for the whole cohort was 330 (220–590) ng/mL, which was quite close to that of patients with stable coronary artery disease [3, 26]. Notably, the reported D-dimer levels of both the current and previous studies are generally lower than 500 ng/mL [3, 4, 12, 14, 15], the threshold for diagnosing deep vein thrombosis, which means that the thrombus burden within the coronary artery poses limited impacts on the systemic D-dimer level in the context of ACS. However, the risk of adverse events has already begun to increase at such low levels of D-dimer. In a research by Simes et al., the risk of all-cause death increases for 59 % in coronary heart disease patients with D-dimer higher than 273 ng/mL [4]. The current results showed that relative risk for all-cause death increased for 58 and 98 % for patients with median (420–1150 ng/mL) and high level (≥ 1150 ng/mL) of D-dimer, respectively, suggesting that the risk of death was elevated even though the absolute value of D-dimer was below the traditional threshold. Similar findings were observed for other secondary endpoints, which affirmed the associations between outcomes and D-dimer. Based on these findings, D-dimer was more of a reflection for the systemic prothrombotic states, which therefore predisposed ACS-PCI patients for long-term adverse events. Moreover, the association between D-dimer and outcomes remained stable after adjustment for various risk factors, including antegrade blood flow. This suggested that the linkage between adverse outcomes and D-dimer elevation was not completely mediated by the failure of reperfusion. In sum, our findings supported an independent association between D-dimer and adverse events. To our knowledge, the current study acquired the largest sample size of its kind, and was also the first to depict a dose-response association between D-dimer and various outcomes.

### Additional prognostic insight from D-dimer level

Although there have been many studies suggesting a linkage between fibrinolytic components and adverse events, D-dimer is generally considered to be unspecific, and not included for the risk stratification in routine clinical practice [1, 5–7]. Meanwhile, few studies have evaluated prognostic value of D-dimer when incorporated to commonly used risk tools for ACS patients. The current study showed that the addition of D-dimer levels (both as continuous and categorial variables) improved the accuracy of risk predictions for all-cause death. D-dimer not only improved the C-index for models of traditional risk factors, but also the clinical risk scores. Because D-dimer remained an independent risk factor when adjusted for various clinical characteristics, D-dimer might provide information beyond demographics, comorbidities, and hemodynamics. The interaction between coagulation system and blood vessels is one of the major interpretations. Recent studies show D-dimer and fibrin degradation products are associated to unstable features of coronary plaques (e.g., greater plaque burden, larger area of necrotic core or calcification, and less fibrotic components) [8, 9]. Activities and exposure of these sub-endothelial contents could directly activate the coagulation cascade [27–30], which constitutes a prothrombotic and inflammatory state leading to restenosis and ischemia [11, 31, 32]. Moreover, D-dimer has been shown to associate with various complications after ACS, like no-reflow and heart failure, which could be attributed to pre-existing massive thrombus within the coronary artery, multifocal vessel wall-related fibrin formation, and a systemic prothrombotic state [16, 33, 34]. Therefore, the level of D-dimer reflected the collective prognostic impacts from the activation of the coagulation system. As current risk models for ACS patients (e.g., GRACE score, TIMI score) generally predict outcomes based on clinical characteristics and hemodynamics [22, 24], the addition of D-dimer might have backed up these models by providing extra information on prothrombotic tendency and vulnerable lesions. In case of D-dimer elevation, the need of more intensive revascularization and antithrombotic treatments should be carefully considered, in order to achieve long-term coronary patency and low risk of recurrent ischemia.

### Clinical applications of D-dimer

Although D-dimer level has been reported to correlate with outcomes, the thresholds to define the high-risk population is so far unclear and not validated [4, 8, 12–15, 17]. Therefore, D-dimer is seldom used in clinical practice to assess thrombotic risk, although it is one of the most easily accessed biomarkers for thrombosis. Considering the constant does-response association between D-dimer and outcomes, we used the X-tile program to look for the optimal cut points, and found that the thresholds of 420 ng/mL and 1150 ng/mL could effectively differentiate patients with low, median, and high risk of all-cause death. Stepwise increase of mortality was observed for both the predictions and observations, which showed favorable accuracy and correlation. This simple and clear risk stratification based on D-dimer level could have the potential to prompt for more tailored risk stratifications and treatments. For patients with high D-dimer (≥ 1150 ng/mL), nearly one in ten patients died within two years, while over 20 % of patients died within five years, indicating the unmet need of risk factors control and treatments. For patients of this kind, more intensive antithrombotic medications are reasonable choices to reduce the risk of stent thrombosis and recurrent ischemia [35, 36], including the use of more potent P2Y12 inhibitors and low dose of oral anticoagulants. More intensive revascularizations (e.g., complete revascularization) should be considered to further lower the risk of death and ischemia [37]. For patients with median level of D-dimer (420–1150 ng/mL), the intermediate outcomes also suggested a possible need for more intensive treatments, but further examinations and evaluations should be performed to validate whether there are strong indications. In this scenario, intravascular imaging examinations should be considered for assessing the stability of culprit and non-culprit arteries, which might help decision making on revascularization strategies [8, 9]. P2Y12 gene tests would be beneficial to further differentiate non-responders for clopidogrel, and prompt the switch for more potent P2Y12 inhibitors to further lower the risk of ischemia [38]. Other risk factors (e.g., hypertension, diabetes, lifestyles) should also be reviewed to see whether there are neglected causes to drive up the level of D-dimer in this intermediate group of patients [39–41]. For patients with low D-dimer (< 420 ng/mL), the one-year mortality was about 1 %, suggesting a generally low risk profile, for which routine care would be adequate to assure a favorable outcome. With a constant association between D-dimer and outcomes, physicians might also consider referring to the dynamic changes of D-dimer, to see whether the current treatments are effective in lowering the thrombotic risk, and quantitatively evaluate how much risk reduction the medication has brought to patients.

### Limitations

The general limitations of this study are as follow. Firstly, patients in this study were retrospectively recruited. Although D-dimer was measured prospectively, residual confounding could exist, and the causality between elevation of D-dimer and outcomes could not be fully defined. Secondly, this observational study was accomplished in a single institute, and external validation was not performed due to limited resources of data. Despite the large sample size and internal validation, extrapolations of conclusions from the current study might require further validation.

## Conclusions

D-dimer was an independent predictor of adverse outcomes for ACS patients treated by PCI. When combined with clinical risk factors, the GRACE score, and the TIMI score, D-dimer significantly improved risk predictions for mortality. Risk stratifications based on D-dimer was feasible to differentiate patients with higher risk of death in both short and long term, which could assist decision making of treating strategies.

## Supplementary information

Additional file 1 **Fig. S1.** Calibration curves for Cox regression models including D-dimer levels (high, median, and low) for all-cause mortality at 180 days. **Fig. S2.** Calibration curves for Cox regression models including D-dimer levels (high, median, and low) for all-cause mortality at 1 year. **Fig. S3.** Calibration curves for Cox regression models including D-dimer levels (high, median, and low) for all-cause mortality at 2 years. **Fig. S4.** Calibration curves for Cox regression models including D-dimer levels (high, median, and low) for all-cause mortality at 5 years. **Fig. S5.** Calibration curves for Cox regression models including D-dimer levels (logarithmic) for all-cause mortality at 180 days. **Fig. S6.** Calibration curves for Cox regression models including D-dimer levels (logarithmic) for all-cause mortality at 1 year. **Fig. S7.** Calibration curves for Cox regression models including D-dimer levels (logarithmic) for all-cause mortality at 2 years. **Fig. S8.** Calibration curves for Cox regression models including D-dimer levels (logarithmic) for all-cause mortality at 5 years.

## Data Availability

The data used to support the findings of this study are available from the corresponding author upon request, but not publicly available, as the institution (Fuwai Hospital) requires all outer accesses to clinical data of patients to be applicated and processed in a case-by-case manner.

## Abbreviations

PCI: percutaneous coronary intervention
ACS: acute coronary syndrome
GRACE score: the Global Registry of Acute Coronary Events risk score
TIMI score: the Thrombolysis in Myocardial Infarction risk score
STEMI: ST-segment elevation myocardial infarction
NSTE-ACS: non-ST-elevation acute coronary syndrome
RCS: restricted cubic spline
