# Supplementary material for: Prognostic Value of D-dimer in patients with acute coronary syndrome treated by percutaneous coronary intervention: a retrospective cohort study
Source: Thromb J. 2021 May 7;19:30. doi: 10.1186/s12959-021-00281-y (PMC8106213; doi:10.1186/s12959-021-00281-y)

**Supplementary figure 1.** Calibration curves for Cox regression models including D-dimer levels (high, median, and low) for all-cause mortality at 180 days.


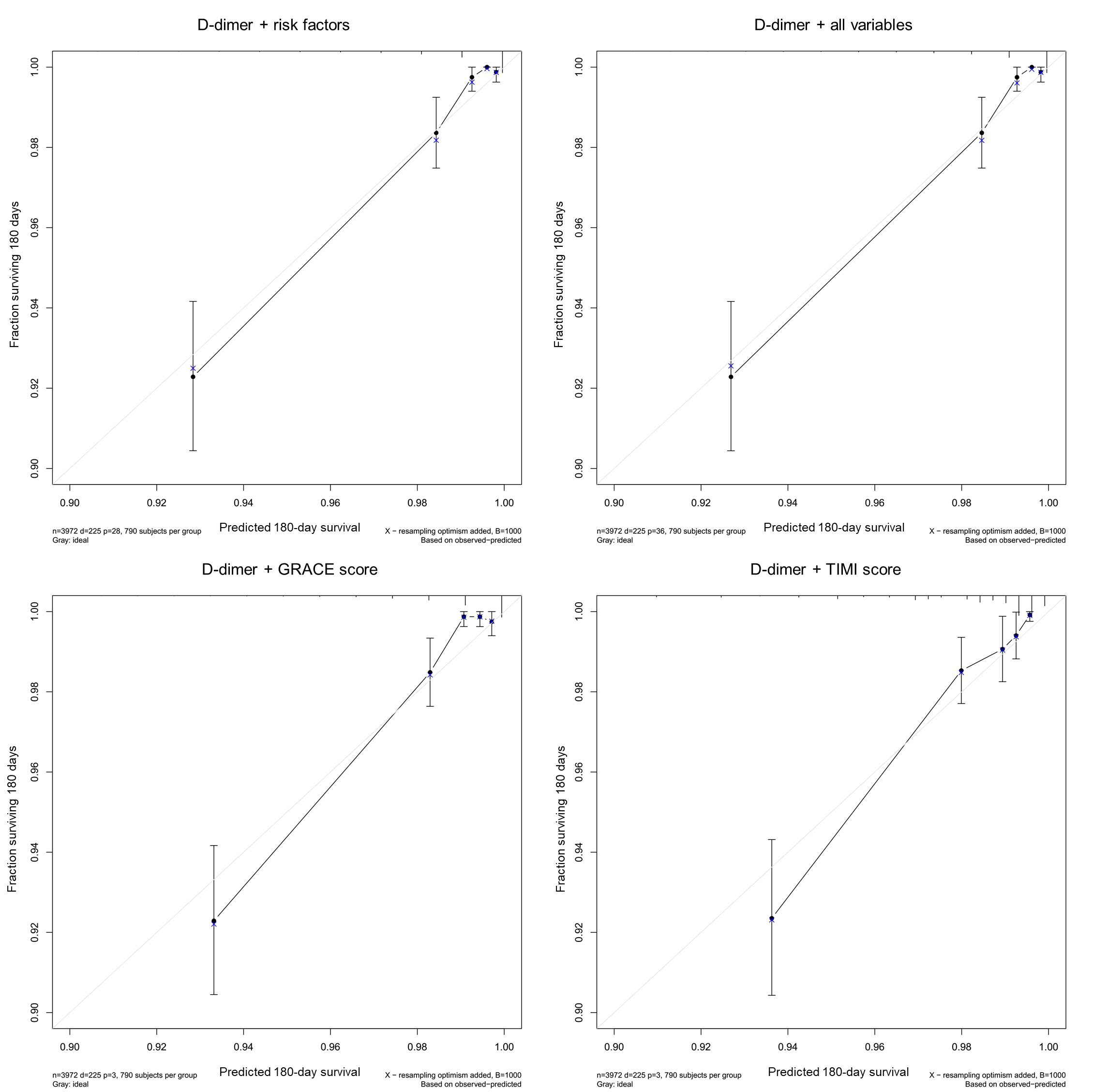


**Supplementary figure 2.** Calibration curves for Cox regression models including D-dimer levels (high, median, and low) for all-cause mortality at 1 year.


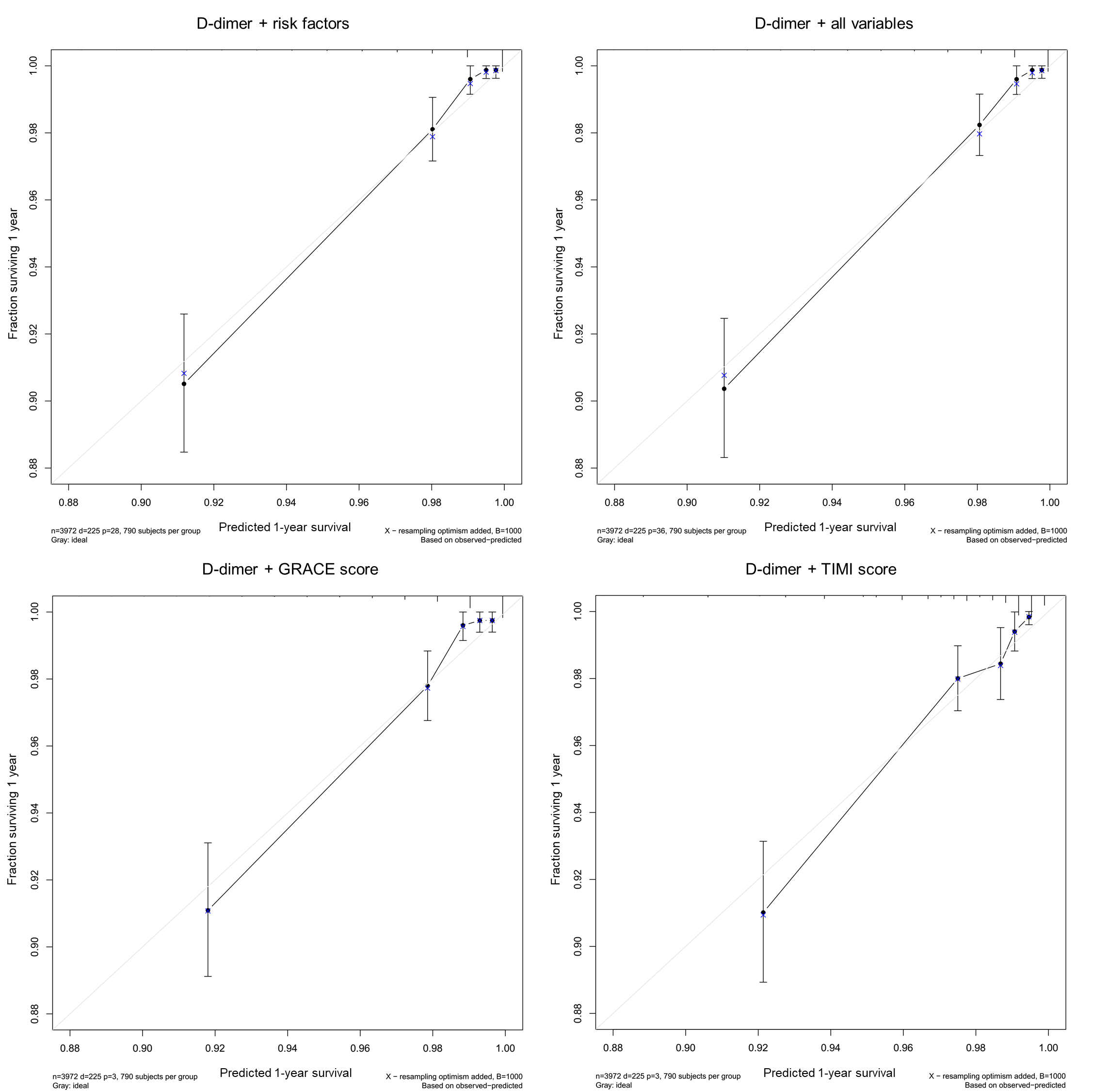


**Supplementary figure 3.** Calibration curves for Cox regression models including D-dimer levels (high, median, and low) for all-cause mortality at 2 years.


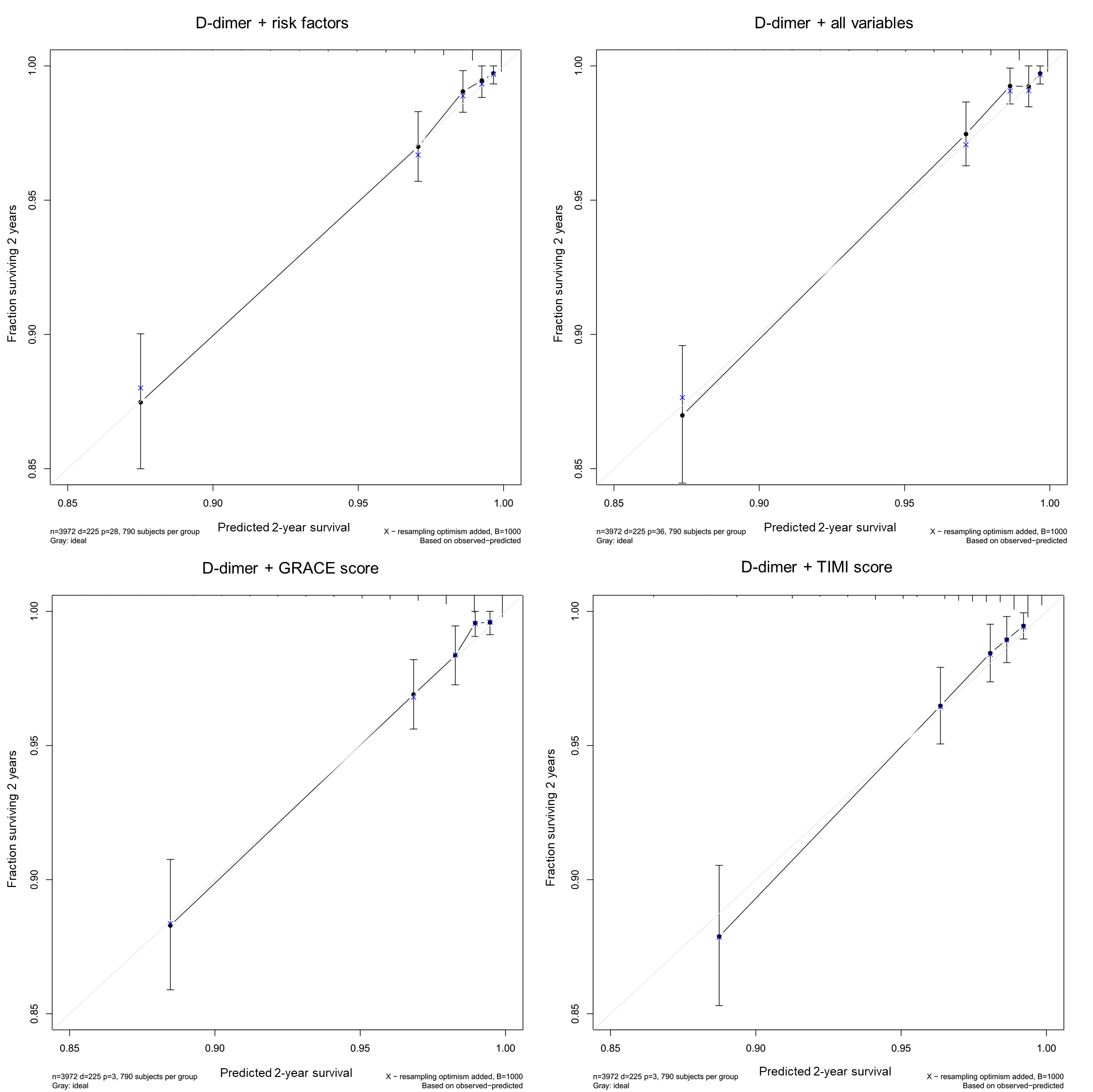


**Supplementary figure 4.** Calibration curves for Cox regression models including D-dimer levels (high, median, and low) for all-cause mortality at 5 years.


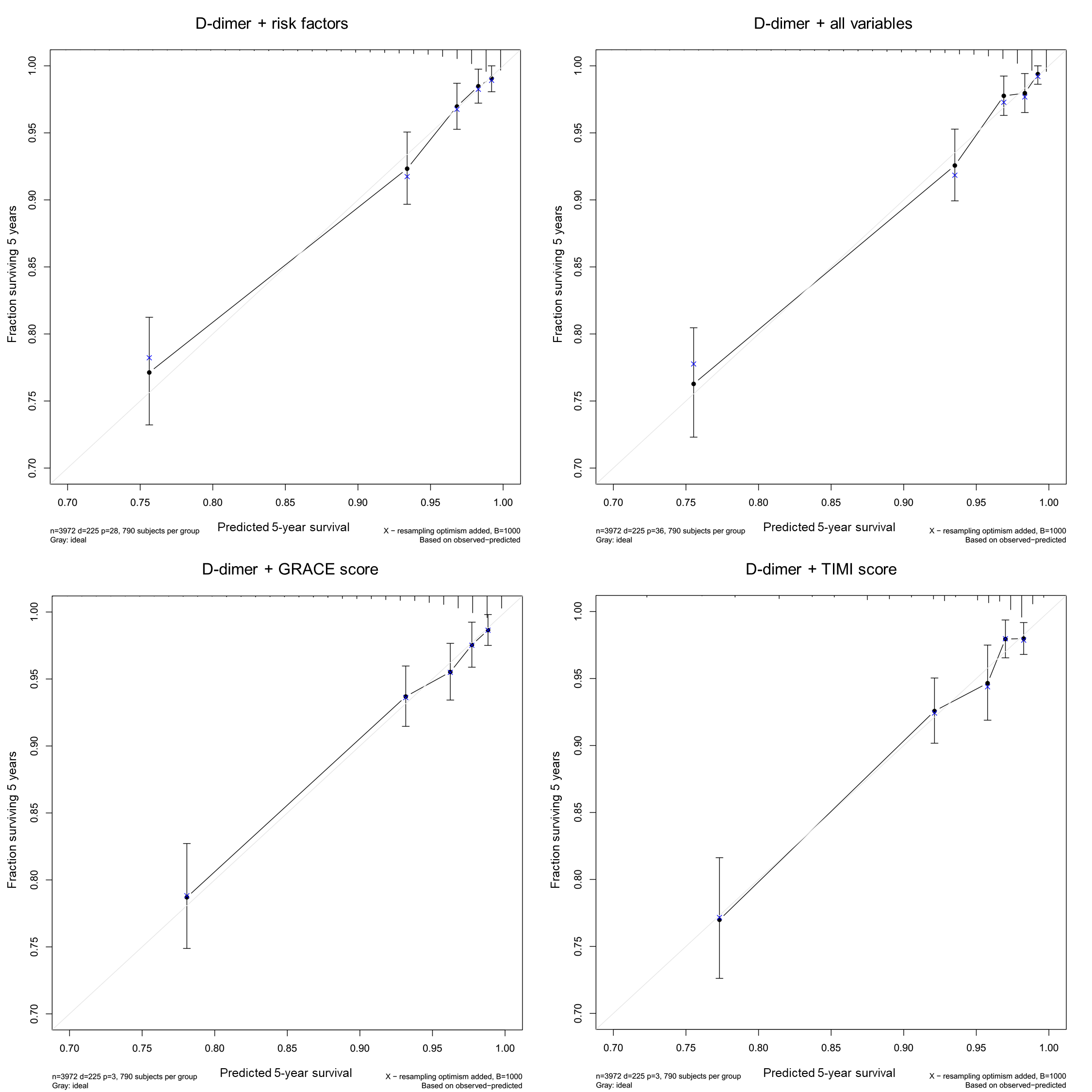


**Supplementary figure 5.** Calibration curves for Cox regression models including D-dimer levels (logarithmic) for all-cause mortality at 180 days.


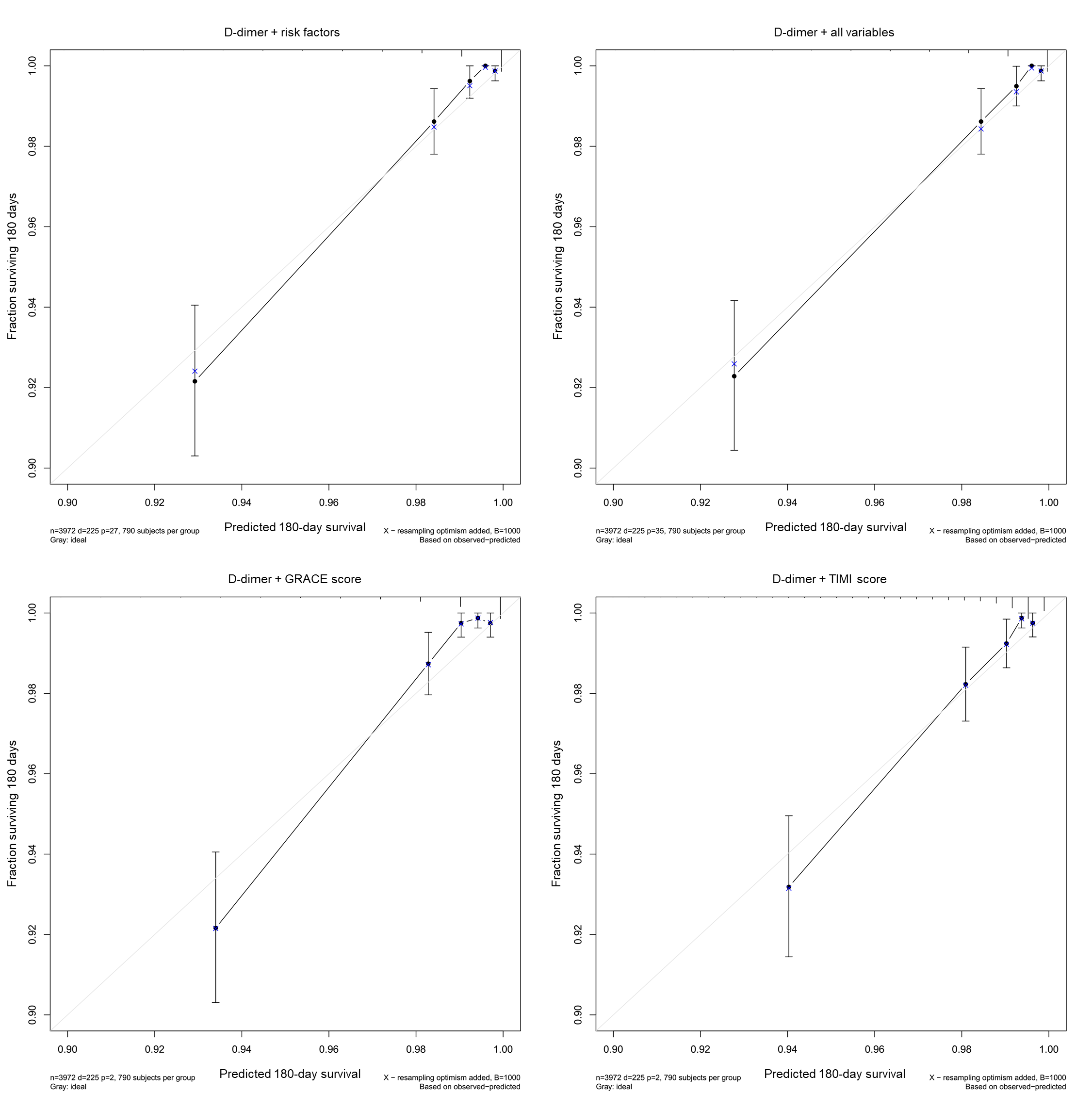


**Supplementary figure 6.** Calibration curves for Cox regression models including D-dimer levels (logarithmic) for all-cause mortality at 1 year.


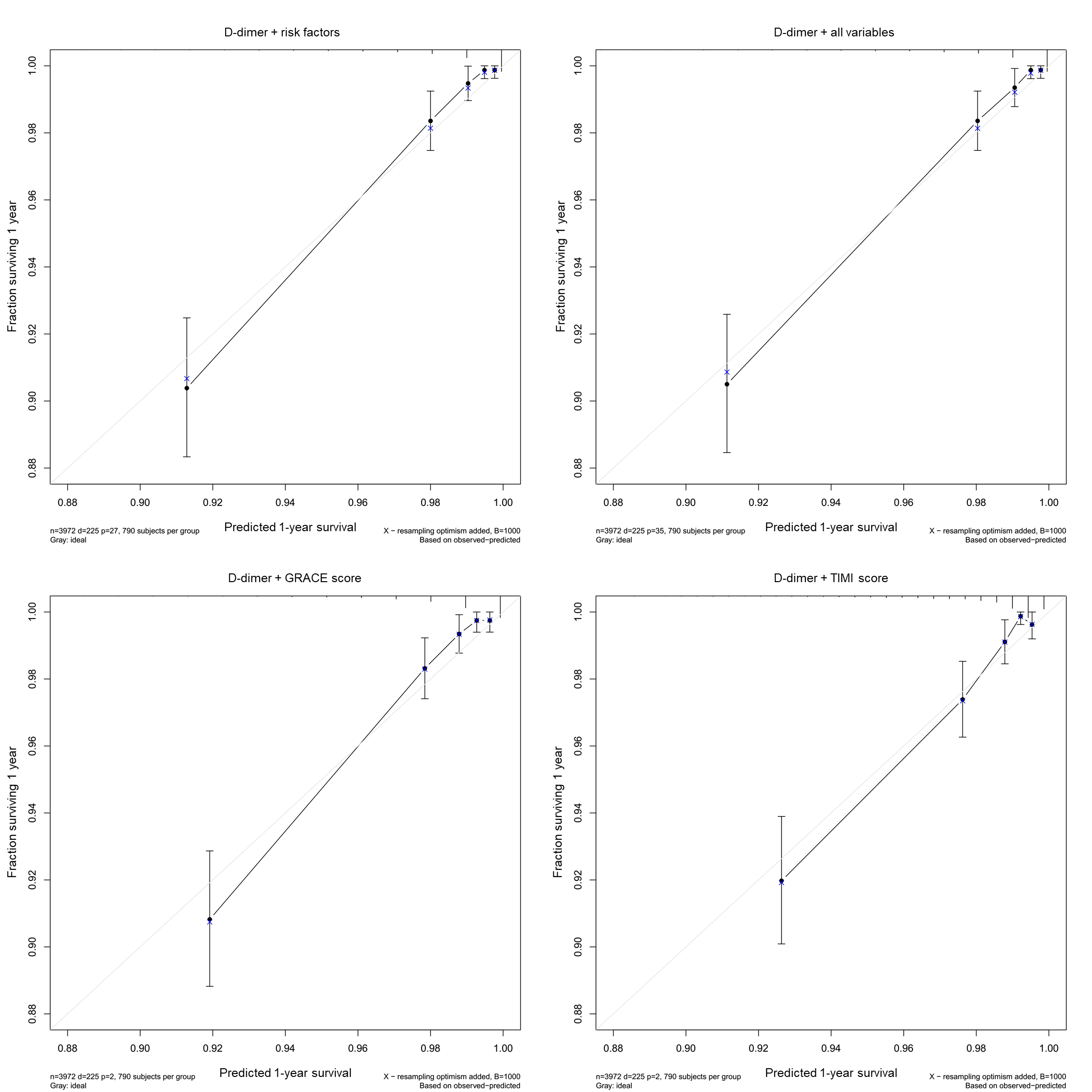


**Supplementary figure 7.** Calibration curves for Cox regression models including D-dimer levels (logarithmic) for all-cause mortality at 2 years.


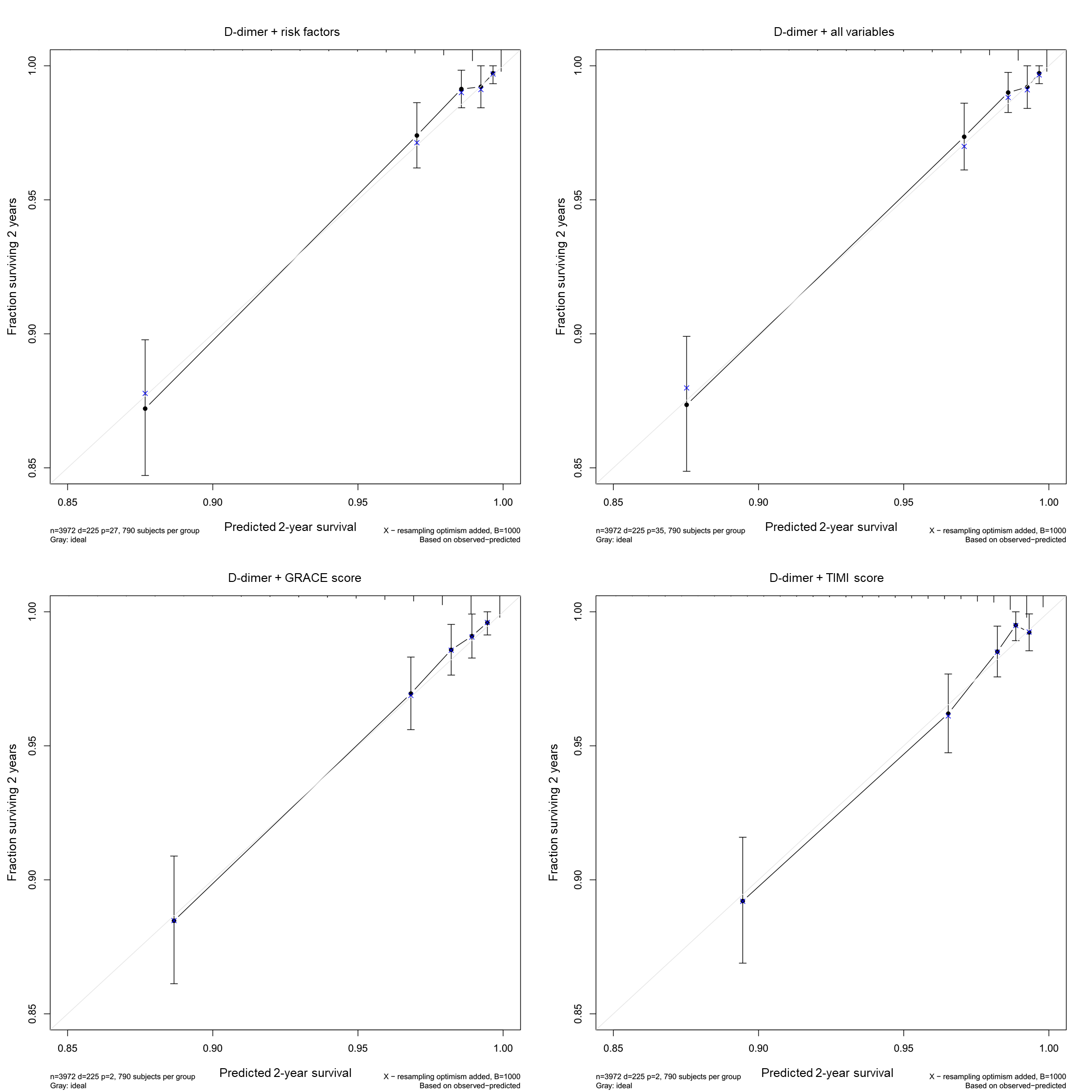


**Supplementary figure 8.** Calibration curves for Cox regression models including D-dimer levels (logarithmic) for all-cause mortality at 5 years.


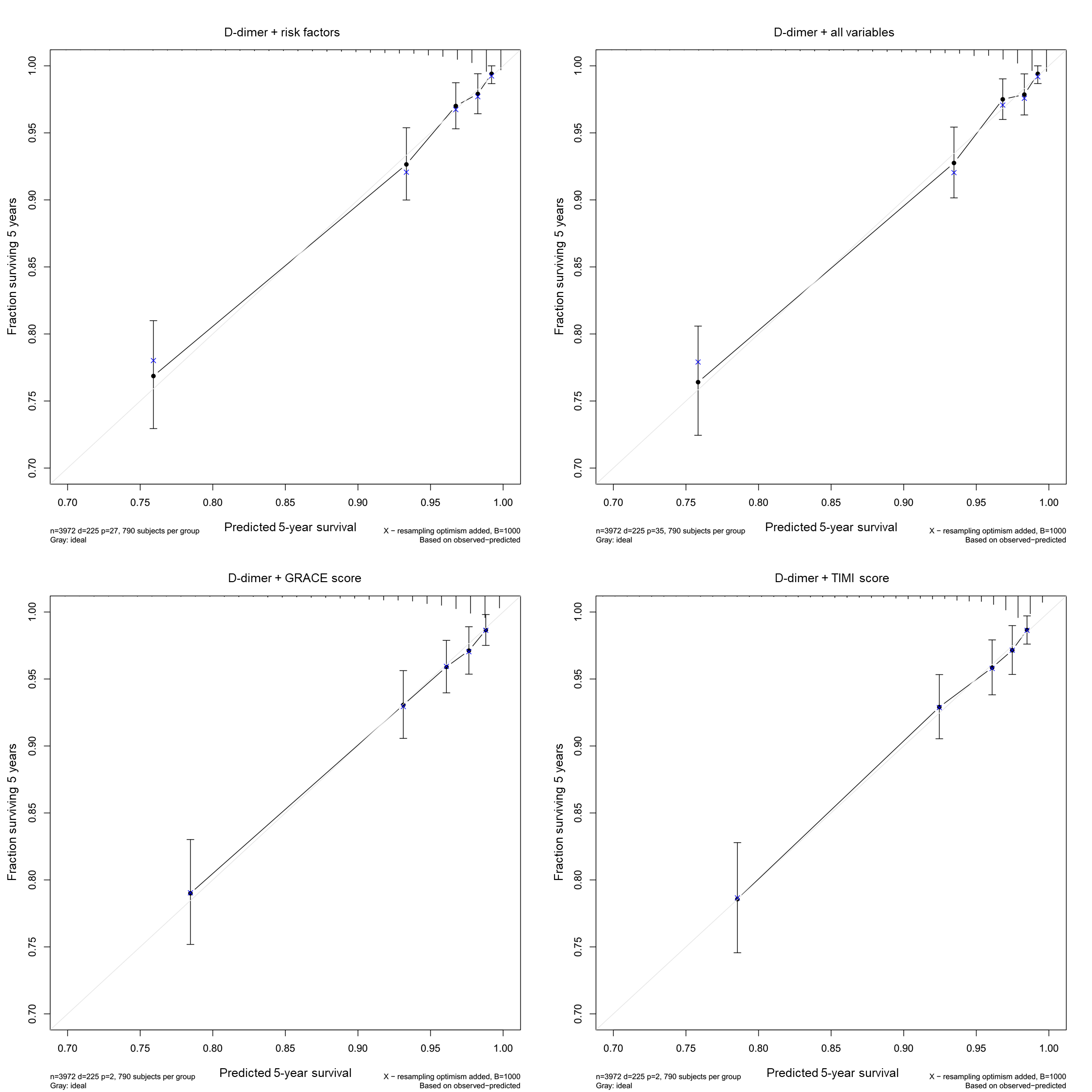

Supplement: Supplementary file 1 — Fig. S1. Calibration curves for Cox regression models including D-dimer levels (high, median, and low) for all-cause mortality at 180 days. Fig. S2. Calibration curves for Cox regression models including D-dimer levels (high, median, and low) for all-cause mortality at 1 year. Fig. S3. Calibration curves for Cox regression models including D-dimer levels (high, median, and low) for all-cause mortality at 2 years. Fig. S4. Calibration curves for Cox regression models including D-dimer levels (high, median, and low) for all-cause mortality at 5 years. Fig. S5. Calibration curves for Cox regression models including D-dimer levels (logarithmic) for all-cause mortality at 180 days. Fig. S6. Calibration curves for Cox regression models including D-dimer levels (logarithmic) for all-cause mortality at 1 year. Fig. S7. Calibration curves for Cox regression models including D-dimer levels (logarithmic) for all-cause mortality at 2 years. Fig. S8. Calibration curves for Cox regression models including D-dimer levels (logarithmic) for all-cause mortality at 5 years. [file 12959_2021_281_MOESM1_ESM.docx]
